# Topics of Public Interest

**Published:** 1907-01

**Authors:** 


### TOPICS OF PUBLIC INTEREST.

### The Isthmian Canal.

**The President's Inspection of the Canal Zone. Extracts from his Special Message to Congress reporting the Isthmian Status.**

PRESIDENT ROOSEVELT spent three days in the Isthmian canal zone in the month of November, 1906, during which he worked from twelve to eighteen hours a day, familiarising himself with the conditions incident to and requisite for the digging of the canal.

The President has added another to his already many-sided qualities. He is a great inspector. He inspected the hospitals and the white and colored patients; the zone police, examining the men individually; the schools and school children, white and colored; the city of Panama, its streets and water supply; the digging with the steam shovels, including the drilling and blasting as well as the dirt trains; the buildings in the different villages, including accommodations for the employees, married and unmarried, white and colored; the commissary and quartermasters's stores; the bath houses, cook sheds for the colored laborers and the government hotels; the machine shops; and the conditions at Colon, including the great reservoir back of Mount Hope.

During these three days he talked with scores of different men,—superintendents and heads of departments, divisions and bureaus; steam shovel men, machinists, conductors, and engineers; clerks, health officers, and wives of American employees; colored laborers, and the attendants and managers of commissary stores where food is sold to the colored employees. He had interviews with the British and French consuls, received deputations of machinists and railway men and listened to complaints, suggestions and other expressions; and, finally, inspected the fire department of Colon, besides giving attention to many other minor details, riding over the different portions of the scene of operations at Culebra Cut, the Gatun, La Boca, and Sosa dams. He lunched with engineers, laborers and employees at totally unexpected times; he inspected the marines and talked with citizens. He also attended two receptions, one given by the President of the Republic of Panama, and another by the American employees.

It is probable that never before was an inspection made of so many men, so much material, and covering such an area in so short a time or under such difficult circumstances. The rainy season was on and the downpour was excessive during the entire three days. This record certainly establishes President Roosevelt's title as the great Inspector General. And he is an inspector who inspects.

We are concerned chiefly at this time, however, with the problem of sanitation, and we let the message speak for itself on the subject, the following being the sections relating thereto:

SUCCESSFUL SANITATION.

The first great problem to be solved, upon the solution of which the success of the rest of the work depended, was the problem of sanitation. This was from the outset under the direction of Dr. W. C. Gorgas, who is to be made a full member of the commission if the law as to the composition of the commission remains unchanged. It must be remembered that his work was not mere sanitation as the term is understood in our ordinary municipal work. Throughout the zone and in the two cities of Panama and Colon, in addition to the sanitation work proper, he has had to do all the work that the Marine Hospital Service does as regards the nation, that the health department officers do in the various states and cities, and that Col. Waring did in New York when he cleaned the streets. The results have been astounding. The isthmus had been a byword for deadly unhealthfulness. Now, after two years of our occupation, the conditions as regard sickness and the death rate compare favorably with reasonably healthy localities in the United States. Especial care has been devoted to minimizing the risk due to the presence of those species of mosquitoes which have been found to propagate malarial and yellow fevers. In all the settlements, the little temporary towns or cities composed of the white and black employes, which grow up here and there in the tropic jungle as the needs of the work dictate, the utmost care is exercised to keep the conditions healthy. Everywhere are to be seen the drainage ditches which in removing the water have removed the breeding places of the mosquitoes, while the whole jungle is cut away for a considerable space around the habitations, thus destroying the places in which the mosquitoes take shelter. These drainage ditches and clearings are in evidence in every settlement, and, together with the invariable presence of mosquito screens around the piazzas and of mosquito doors to the houses, not to speak of the careful fumigation that has gone on in all infected houses, doubtless explain the extraordinary absence of mosquitoes. As a matter of fact, but a single mosquito, and this not of the dangerous species, was seen by any member of our party during my three days on the isthmus. Equal care is taken by the inspectors of the Health Department to secure cleanliness in the houses and proper hygienic conditions of every kind. I inspected between twenty and thirty water-closets, both those used by the white employes and those used by the colored laborers. In almost every case I found the conditions perfect. In but one case did I find them really bad. In this case, affecting a settlement of unmarried white employes, I found them very bad indeed, but the buildings were all inherited from the French company and were being used temporarily while other buildings were in the course of construction, and right near the defective watercloset a new and excellent closet, with a good sewer pipe, was in process of construction and nearly finished. Nevertheless, this did not excuse the fact that the bad condition had been allowed to prevail. Temporary accommodations, even if only such as soldiers use when camped in the field, should have been provided. Orders to this effect were issued. I append the report of Dr. Gorgas on the incident. I was struck, however, by the fact that in this instance, as in almost every other where a complaint was made which proved to have any justification whatever, it appeared that steps had already been taken to remedy the evil complained of, and that the trouble was mainly due to the extreme difficulty and often impossibility, of providing in every place for the constant increase in the numbers of employes. Generally the provision is made in advance, but it is not possible that this should always be the case; when it is not there ensues a period of time during which the conditions are unsatisfactory, until a remedy can be provided; but I never found a case where the remedy was not being provided as speedily as possible.

### HOSPITALS AND THEIR TREATMENT.

I inspected the large hospitals at Ancon and Colon, which are excellent examples of what tropical hospitals should be. I also inspected the receiving hospitals in various settlements. I went through a number of the wards in which the colored men are treated, a number of those in which the white men are treated—Americans and Spaniards. Both white men and black men are treated exactly alike, and their treatment is as good as that which could be obtained in our first class hospitals at home. All the patients that I saw, with one or two exceptions, were laborers or other employes on the canal works and railways, most of them being colored men of the ordinary laborer stamp. Not only are the men carefully cared for whenever they apply for care, but so far as practicable a watch is kept to see that if they need it they are sent to the hospitals, whether they desire to go or not. From no responsible source did any complaint come to me as to the management of the hospital service, although occasionally a very ignorant West India Negro when he is first brought into the hospital becomes frightened by the ordinary hospital routine.

### HEALTH SHOWING REMARKABLY GOOD.

Just at present the health showing on the isthmus is remarkably good—so much better than in most sections of the United States that I do not believe that it can possibly continue at quite its present average. Thus, early in the present year a band of several hundred Spaniards were brought to the isthmus as laborers, and additions to their number have been made from time to time; yet, since their arrival in February last, but one of those Spaniards thus brought over to work on the canal has died of disease, and he of typhoid fever. Two others were killed—one in a railroad accident, and one by a dynamite explosion. There has been for the last six months a well nigh steady decline in the death rate for the population of the zone, this being largely due to the decrease in deaths from pneumonia which has been the most fatal disease on the isthmus. In October there were ninety-nine deaths of every kind among the employes of the isthmus. There were then on the rolls 5,500 whites, seven-eighths of them being Americans. Of these whites but two died of disease, and as it happened neither man was an American. Of the 6,000 white Americans, including some 1,200 women and children, not a single death has occurred in the last three months, whereas in an average city in the United States the number of deaths for a similar number of people in that time would have been about thirty from disease. This very remarkable showing cannot, of course, permanently obtain, but it certainly goes to prove that if good care is taken the isthmus is not a particularly unhealthy place. In October, of the 19,000 Negroes on the roll, eighty-six died from disease, pneumonia being the most destructive disease and malarial fever coming second. The difficulty of exercising a thorough supervision over the colored laborers is of course greater than is the case among the whites, and they are also less competent to take care of themselves, which accounts for the fact that their death rate is so much higher than that of the whites, in spite of the fact that they have been used to similar climatic conditions. Even among the colored employes it will be seen that the death rate is not high.

DIMINUTION OF MOSQUITOES.

In Panama and Colon the death rate has also been greatly reduced, this being directly due to the vigorous work of the special brigade of employes who have been inspecting houses where the stegomyia mosquito is to be found, and destroying its larvæ and breeding places, and doing similar work in exterminating the malarial mosquitoes—in short, performing all kinds of hygienic labor. A little over a year ago all kinds of mosquitoes, including the two fatal species, were numerous about the Culebra cut. In this cut during last October every room of every house was carefully examined, and only two mosquitoes, neither of them of the two fatal species, were found. Unfaltering energy in inspection and in disinfecting and in the work of draining and of clearing brush are responsible for the change. I append Dr. Gorgas's report on the health conditions; also a letter from Surgeon General Rixey to Dr. Gorgas. The Surgeon General reported to me that the hygienic conditions on the isthmus were about as good as, for instance, those in the Norfolk Navy Yard.

Corozal, some four miles from La Boca, was formerly one of the most unsanitary places on the isthmus, probably the most unsanitary. There was a marsh with a pond in the middle. Dr. Gorgas had both the marsh and pond drained and the brush cleared off, so that now, when I went over the ground, it appeared like a smooth meadow, intersected by drainage ditches. The breeding places and sheltering spots of the dangerous mosquitoes had been completely destroyed. The result is that Corozal for the last six months (like La Boca, which formerly also had a very unsanitary record), shows one of the best sick rates in the zone, having less than 1 per cent. a week admitted to the hospital. At Corozal there is a big hotel filled with employes of the Isthmian Canal Commission, some of them with their wives and families. Yet this healthy and attractive spot was stigmatised as a "hog wallow" by one of the least scrupulous and most foolish of the professional scandal mongers who from time to time have written about the commission's work.

